# An unusual location of retroperitoneal epidermoid cyst in a child: case report and a review of the literature

**DOI:** 10.1186/1757-1626-2-9397

**Published:** 2009-12-24

**Authors:** Nexhmi Sh Hyseni, Sadik S Llullaku, Defrim H Koçinaj, Hysni J Jashari, Baton Z Kelmendi

**Affiliations:** 1 Department of Pediatric Surgery, University Clinical Centre, Rr. e Spitalit p.n, 10.000 Prishtina, Kosovo

## Abstract

We report the case of a 4-year-old girl presenting with the retroperitoneal epidermoid cyst. The lesion presented as an intra-abdominal cyst on physical examination and was followed up with more specific investigations by ultrasound and computed tomographic scanning. The final diagnosis was obtained only after laparotomy where the cystic mass was completely excised and pathological examination was done. The patient is well at 3-year follow-up. epidermoid cyst of the reteroperitoneal space, although rare, should be considered in the differential diagnosis of incidentally discovered intra-abdominal cysts during investigation of irrelevant illnesses or during routine abdominal ultrasound scan.

## Introduction

Retroperitoneal cysts are the least common of abdominal lymphatic malformations and they can be located anywhere in the retroperitoneum. Retroperitoneal cysts should be considered a different entity from mesenteric, omental, splenic and enteric duplication cysts even though they present clinically in a similar fashion [1]. Epidermoid cysts that develop in the retroperitoneal space are quite rare [2]. It may be diagnosed antenatally on prenatal ultrasonography but they usually are discovered incidentally during investigation of irrelevant illnesses or during routine abdominal ultrasound scan [3]. Retroperitoneal cysts are mostly asymptomatic and, if present symptoms, are quite unspecific symptoms depending on the size, location, and complications, such as, haemorrhage, infection, or rupture [4].

Histopathologic examination of a retroperitoneal epidermoid cyst showed a cyst to be lined with squamous stratified epithelium. The wall of the cyst consisted of dense fibrous tissue [2]. Surgical resection is indicated to establish a diagnosis and prevent eventually complications.

## Case presentation

**Figure 1:**
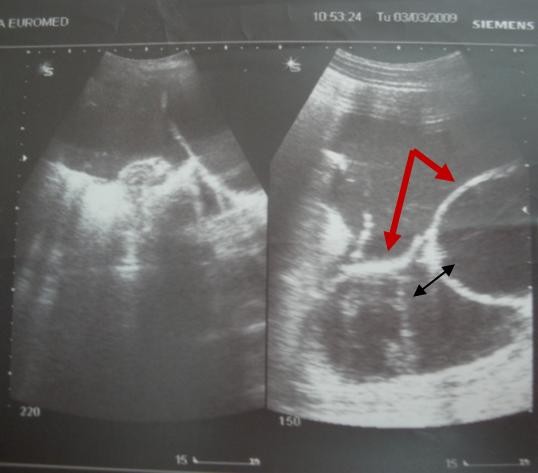
**Ultrasound evaluation typically shows a thin-walled cystic mass (thick arrow), with fine internal septa (thin arrow)**.

**Figure 2:**
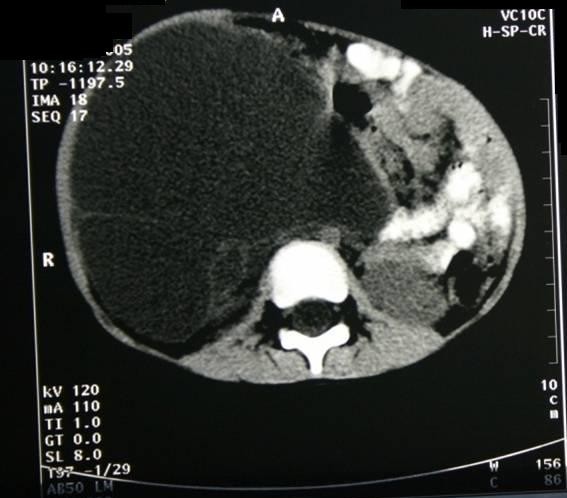
**CT scan shows a well-defined retroperitoneal thin-walled, multilocular cystic mass with septa and fluid attenuation**.

**Figure 3:**
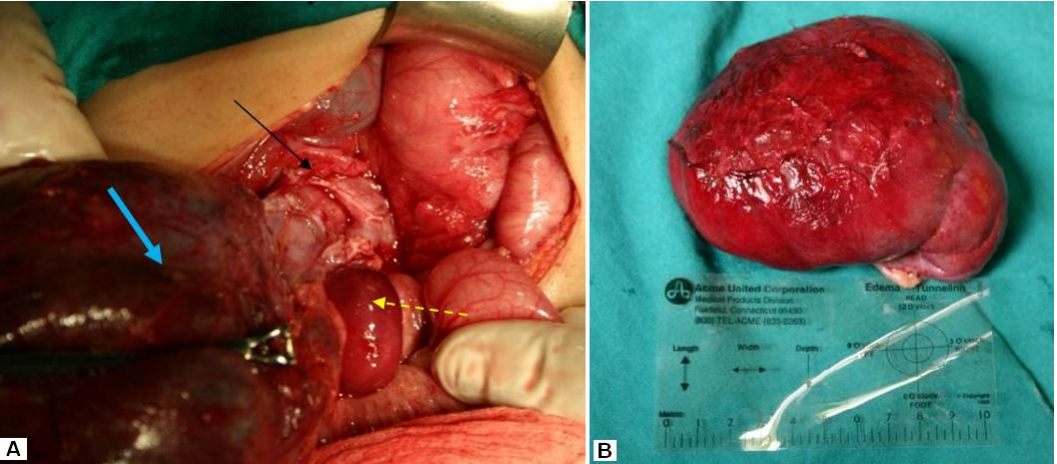
**(a) Intraoperative view on gross inspection of the cystic mass (thick arrow)**. A huge retroperitoneal mass closely connected to the inferior vena cava, and to the homolateral kidney (thin arrow). **(b) **Operative specimen showing giant retroperitoneal cystic mass about 11 cm in greatest dimensions.

**Figure 4:**
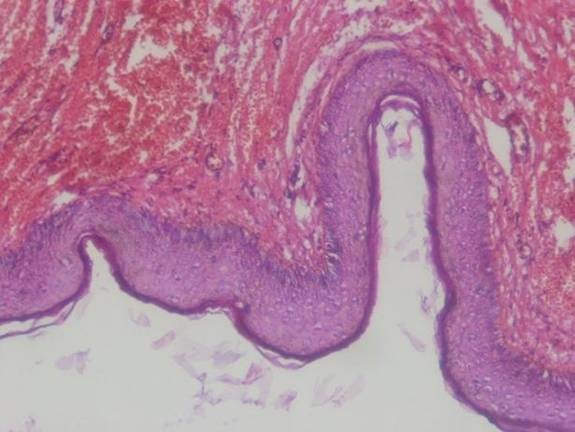
**Photomicrograph shows fibrous tissue lined by stratified squamous epithelium that contains keratinous materials**.

We report the case of a 4-year-old girl, Kosovo Albanian nationality, with a 4-month history of recurrent abdominal pain and abdominal distension without vomiting. At about the same time his parents became aware of an enlargement of the right side of his abdomen. Examination disclosed a soft, non-tender a palpable abdominal mass in the right upper quadrant of the abdomen that was dull to percussion. Laboratory tests were within the normal limits (WBC, 9.3/μl with 64% neutrophils; hemoglobin (9.9 g/100 ml), platelets (2,000,000/μl), except for the erythrocyte sedimentation rate (78 mm/h). The CRP was elevated with a mean count of 104 mg/l. Serological test for hydatid was negative. Alpha-fetoprotein (αFP) level was normal. In an abdominal ultrasound scan revealing a large mass with a thin-walled cystic mass, with fine internal septa, measuring 8 × 7 × 4 cm and was located in the abdomen (Figure 1). CT scan shows a well-defined retroperitoneal thin-walled, multilocular cystic mass with septa and fluid attenuation beginning in the sub-hepatic region displacing the hepatic flexure and proximal transverse colon inferiorly. No bowel obstruction is evident (Figure 2). The child underwent right transversal supraumbilical laparotomy. At operation was found a large retroperitoneal cyst arising from the mid-transverse colon which contained almost a 500 ml of dark brown fluid. A huge retroperitoneal mass 11 × 9 × 7 cm in diameter -closely connected to the inferior vena cava, and to the homolateral kidney (Figure 3a). The cyst was freed intra-abdominally, paying special attention to inferior vena cava, kidney vessels and ureter. No enlarged lymph nodes were detected. Grossly, the cyst was about 11 cm in greatest dimensions (Figure 3b). After excision of the lesion the pathology report confirmed the diagnosis of epidermoid cyst (Figure 4). The postoperative period was uneventful. The patient is now 8 years old, and after the 3-year follow-up, the child was well and free of symptoms without recurrence.

## Discussion

The presence of a retroperitoneal cystic mass in children presents a diagnostic problem which frequently remains undiagnosed until surgery reveals the true nature of the mass. Cysts of the mesentery, retroperitoneum and omentum are uncommon and present with similar incidence, varying between, and 1 per 100.000 hospital admissions in most series [5]. Mesenteric, omental, and retroperitoneal cysts are considered together because of the shared embryologic origin of the structures from which they originate [6,7].

Retroperitoneal cysts should be considered a different entity from mesenteric cysts even though they present clinically in a similar fashion. Epidermoid cysts are slow growing, formed by desquamation of epithelial cells [4]. Epidermoid cyst localized to the retroperitoneum is uncommonly encountered clinical entities. Subdiaphragmatic epidermoid cyst was reported in an 11-Year-old boy, arising from the diaphragmatic muscle [8].

Fakhir et al. [9], and Midorikawa et al. [10], described giant pelvic retroperitoneal epidermoid cyst, and presacral epidermoid cyst, both of adult cases.

To our knowledge, retroperitoneal epidermoid cysts, in this unusual location in children have not been reported in the literature. In our patient, a huge retroperitoneal mass - 11 × 9 × 7 cm in diameter was -closely connected with transverse and ascending colon to the inferior vena cava, and homolateral kidney. In addition to epidermoid retroperitoneal cysts, differential considerations include cystic lesions such as abdominal cystic lymphangioma that occur most commonly in the mesentery of the small bowel, with the retroperitoneum being the second most frequent site [11], Other cystic retroperitoneal mass that may be present are mucinus cystadenoma [12], retroperitoneal bronchogenic cysts [13], and retroperitoneal of gastro duodenal duplication cysts [5]. Cysts that are present in the mesentery, omentum and retroperitoneum are comparable embryologically and pathologically [11]. Imaging with ultrasonography and conventional computed tomography can determine the lesion as cystic whether they are complex or simple, unilocular or multilocular, even though ultrasound characteristics of epidermoid cysts are not different from mesenteric and omental cysts [14,2]. In our case, initially a retroperitoneal hypoechogenic mass was detected by sonography. The US and CT images was not specific enough to allow differentiation of epidermoid cysts from other retroperitoneal cystic masses. The examination that confirms the diagnosis is an explorative laparotomy and histopathological examination. At histological analysis, retroperitoneal epidermoid cyst lesion has a component of stratified squamous epithelium. Epidermoid cyst or epithelial cysts have an epithelial lining and are thus true cysts. Lining produces fully matured, keratinized cellular debris, which fill the cavity of the cyst [8].

Symptoms are most frequently caused by the compressive effect of the cyst on surrounding structures and rarely by complications of the cyst: inflammation, abscess, and rupture.

Our patient become last 4 months symptomatic, resulting from local mass effect causing flank pain, and enlargement of the right side of his abdomen. In our cases surgical approach was successful in removing the completely cyst tissue mass without recurrence. The incidence of recurrence for retroperitoneal cysts is higher than with other forms of cysts because their proximity to major blood vessels and vital structures sometimes makes them difficult to completely excise [15]. In our cases cystic mass was extended closely with vena cava and renal vessels and complete removal was done without difficulty

## Conclusion

One case of large retroperitoneal epidermoid cyst an unusual location is presented; the finding of a right upper quadrant retroperitoneal mass in a child should lead the physician to a prompt assessment and diagnosis of possible retroperitoneal epidermoid cyst. Successful treatment dependent upon early diagnosis, careful operative technique through abdominal incision, and adequate preoperative and postoperative management. Management of a retroperitoneal cystic lesion is determined by the underlying pathology. Retroperitoneal cystic lesions, although are benign, can be technically difficult to excise because of the proximity to major vessels or other organs.

## Consent

Written informed consent was obtained from the patient for publication of this case report and accompanying images. A copy of the written consent is available for review by the journal's Editor-in-Chief.

## Competing interests

The authors declare that they have no competing interests.

## Authors' contributions

NSH carried out design of the study, drafted the manuscript, and was a major contributor in writing the manuscript. SSL participated in design of the study and in drafting the manuscript. HJJ performed the literature review, and was involved in writing the manuscript. DHK and BZK were involved in literature and image collection. All authors read and approved the final manuscript.
